# Backbone Engineering of Polythiophenes via Quinoid and Cyano Dual Functionalization for n-Type Polymers

**DOI:** 10.3390/polym18151900

**Published:** 2026-08-03

**Authors:** Weipeng Sun, Yanlin Wei, Peng Wang, Dingqin Hu, Peng Dai, Wenge Zhang, Dian Zhang, Jianfeng Li, Yongqiang Shi, Xugang Guo

**Affiliations:** 1 Anhui Provincial Key Laboratory of Advanced Catalysis and Energy Materials, Anhui Key Laboratory of Optoelectronic Magnetic Functional Complex and Nano Complex, School of Chemistry and Chemical Engineering, Anqing Normal University, Anqing 246133, China; daipeng@aqnu.edu.cn (P.D.); wenggezhang@aqnu.edu.cn (W.Z.); 2 Shenzhen Key Laboratory of Printed Electronics, Department of Materials Science and Engineering, Southern University of Science and Technology, Shenzhen 518055, China; 12432513@mail.sustech.edu.cn (Y.W.); 12332073@mail.sustech.edu.cn (P.W.); dingqihu@cityu.edu.hk (D.H.); 3 Institute of Industrial Science, The University of Tokyo, Komaba 4-6-1, Meguro-ku, Tokyo 153-8505, Japan; dian@iis.u-tokyo.ac.jp; 4 School of Materials Science and Engineering, Shaanxi Normal University, Xi’an 710119, China; lijf0630@snnu.edu.cn; 5 Key Laboratory of Functional Molecular Solids, Ministry of Education, School of Chemistry and Materials Science, Anhui Normal University, No. 189, Jiuhua South Road, Wuhan 241002, China; shiyq@ahnu.edu.cn

**Keywords:** quinoid structure, cyano group, n-type polymer, organic field-effect transistor, organic thermoelectric

## Abstract

Developing high-performance n-type polymer semiconductors is hindered by the scarcity of strong electron-deficient building blocks. Herein, we report a dual-functionalization strategy that integrates both quinoid and cyano groups into polythiophene backbones to construct n-type polymers. Two new polymers, PQTTCN and PQTVTCN, were synthesized via the Stille copolymerization of a thienoquinoid-based dibrominated monomer (TTD2T-Br) with cyano-functionalized bithiophene and thienylene-vinylene-thienylene distannyl monomers, respectively. Electrochemical and computational analyses confirm that both polymers exhibit low-lying LUMO levels of −4.07 eV and highly planar backbones. In organic field-effect transistors, PQTTCN and PQTVTCN show unipolar n-type charge transport, with electron mobilities of 0.036 and 0.002 cm^2^ V^−1^ s^−1^, respectively, which are attributed to their deep frontier molecular orbitals and planar conformations. Upon doping, both polymers exhibit n-type thermoelectric performance, achieving an electrical conductivity and power factor values of 0.16 S cm^−1^ and 0.75 μW m^−1^ K^−2^ for PQTTCN and 0.043 S cm^−1^ and 0.17 μW m^−1^ K^−2^ for PQTVTCN, respectively. AFM and GIWAXS results demonstrate that PQTTCN has better dopant compatibility and higher crystallinity than PQTVTCN. This work highlights that the combination of quinoid and cyano units offers a promising strategy for developing high-performance n-type polymer semiconductors for organic electronics.

## 1. Introduction

Polymer semiconductors have garnered significant attention over the past decade, owing to their potential for mechanically stretchable and solution-processable organic electronics, such as organic field-effect transistors (OFETs) [1,2,3], organic solar cells (OSCs) [4,5], and organic thermoelectrics (OTEs) [6,7]. A wide variety of polymer semiconductors have been developed to improve device performance by tuning their electronic structures, including bandgap, frontier molecular orbital (FMO), and microstructure (i.e., surface morphology, crystallinity, and molecular orientation) [8,9,10]. Recent years have witnessed remarkable progress in p-type (hole-transporting) polymer semiconductors, with electrical conductivities (σ) exceeding 3000 S cm^−1^ [11,12]. In sharp contrast, the overall development of n-type (electron-transporting) polymer semiconductors lags far behind, constrained by the limited availability of electron-deficient building blocks and substantial synthetic challenges [13,14]. As a result, the long-standing performance gap between p-type and n-type materials has continued to widen, underscoring the urgent need for high-performance n-type polymer semiconductors that can match their p-type counterparts.

**Figure 1:**
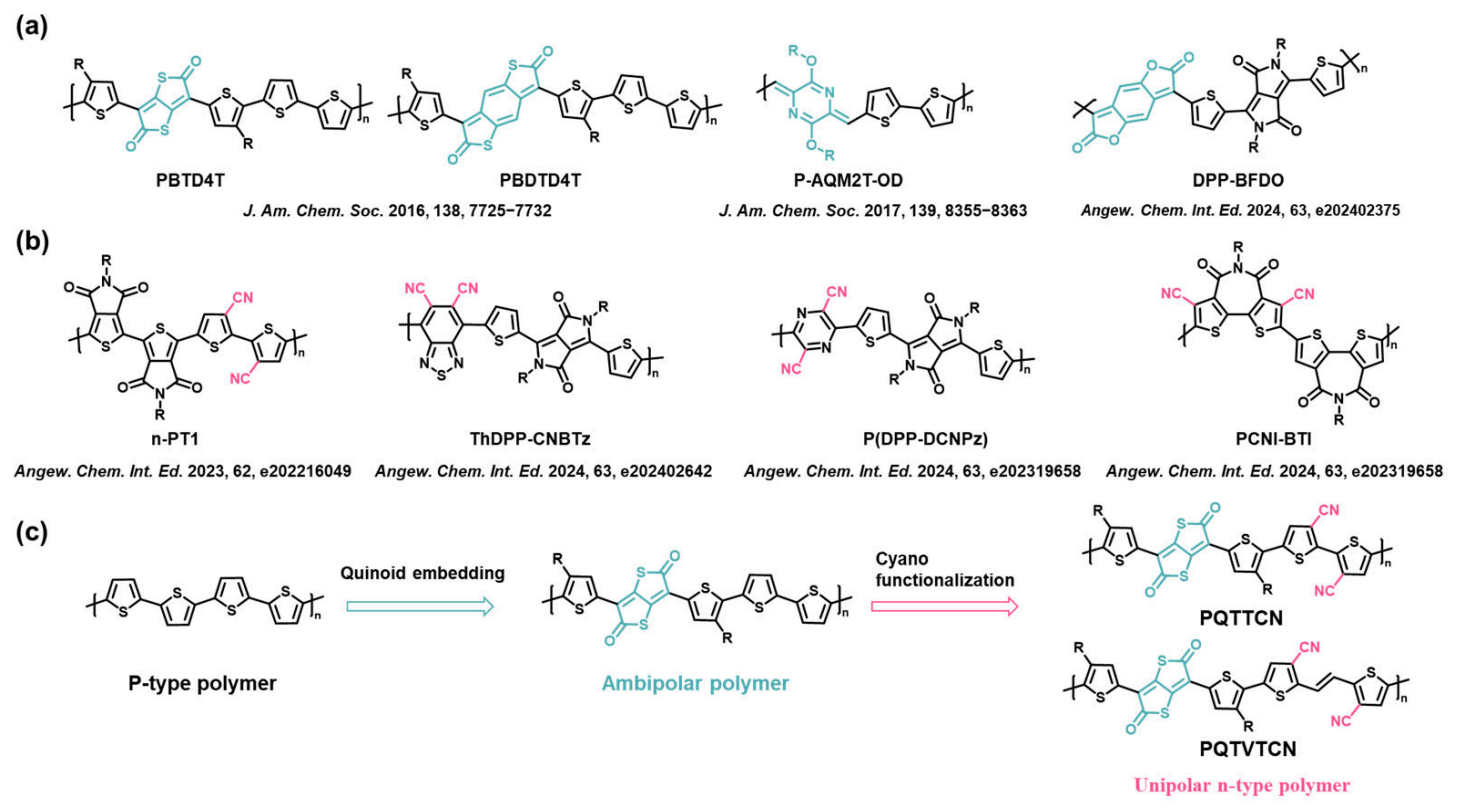
Chemical structures of (**a**) quinoid-based [21,27,28] and (**b**) cyano-functionalized conjugated polymers [30,32,34]; (**c**) design strategy for unipolar n-type polymers with integrated quinoid and cyano dual functionalization.

Among various electron-withdrawing building blocks, quinoid molecules have attracted considerable interest as a promising platform for developing n-type polymer semiconductors [13,15,16]. Compared with conventional aromatic systems, quinoid structures feature strong electron deficiency and enhanced π-orbital delocalization, both of which arise from reduced bond length alternation and a more coplanar conjugated backbone [17,18,19]. Moreover, quinoid-based polymers typically possess low-lying lowest unoccupied molecular orbital (LUMO) levels, which facilitate electron injection and improve n-doping efficiency [20,21]. The deep LUMO levels also impart enhanced ambient stability against water and oxygen, ensuring more stable electron transport under atmospheric conditions [22]. In recent years, considerable progress has been made in the design of quinoid molecules, and their corresponding polymers (Figure 1a) have been increasingly explored for applications in OFETs and OTEs [23,24,25,26]. For example, Takimiya et al. reported two quinoid-based polymers (PBTD4T and PBDTD4T) by incorporating thienoquinoid units into polythiophene backbones [27]. Owing to the electron-deficient nature of these quinoid units, their polymers exhibit deeper LUMO levels than those of unmodified polythiophene, thereby enabling electron transport behavior in OFETs. Liu et al. synthesized a fully planar azaquinodimethane-based polymer (P-AQM2T-OD) due to intramolecular S···N noncovalent interactions, and subsequent optimization of the comonomer units further improved the mobility to over 5 cm^2^ V^−1^ s^−1^ [28,29]. More recently, Huang et al. reported an acceptor–acceptor conjugated polymer (DPP-BFDO) based on a strong electron-deficient benzodifurandione unit [21]. Benefiting from its deep LUMO level, this polymer enables efficient n-type doping, achieving a high σ of 65.68 S cm^−1^. Among these quinoid units, the thieno[3,2-b]thiophene-2,5-dione (TTD) quinoid molecule stands out for its straightforward synthesis, low steric hindrance, and deep LUMO level, and it has consequently been exploited for developing n-type polymers for OFET and OTE applications. Beyond quinoid-based systems, cyano functionalization represents another effective strategy for constructing electron-deficient building blocks, as exemplified by dicyanobithiophene (TTCN) [30,31], dicyanobenzothiadiazole (DCNBT) [32,33], dicyanopyrazine (DCNPz) [34], and cyano-functionalized bithiophene imide (CNI) [35,36]. The corresponding polymers (Figure 1b) exhibit high σ values upon n-type doping, along with unipolar electron transport characteristics. Collectively, these findings demonstrate that incorporating quinoid and cyano moieties into polymer backbones effectively lowers the LUMO levels, thereby enabling both electron injection and n-type doping, which are key prerequisites for high-performance n-type polymer semiconductors.

Given that the structurally simple polythiophene backbone is the most thoroughly explored p-type framework, it is worth considering how it can be directly modified to afford unipolar n-type polymers. To this end, we have designed a dual-functionalization strategy that incorporates electron-withdrawing quinoid and cyano groups into the polythiophene backbone (Figure 1c). The resulting polymers, namely PQTTCN and PQTVTCN, exhibit low-lying LUMO levels (<−4.0 eV) and excellent backbone planarity, both of which are beneficial for achieving n-type polymer semiconductors with high device performance. Accordingly, PQTTCN and PQTVTCN exhibit unipolar electron mobilities (*μ*_e_) of 0.036 and 0.002 cm^2^ V^−1^ s^−1^, respectively. Upon doping with the molecular dopant julolidine-functionalized benzimidazoline (JLBI), both PQTTCN and PQTVTCN display n-type thermoelectric performance. Collectively, these results demonstrate that the dual functionalization with quinoid and cyano groups represents a promising strategy for developing high-performance n-type polymer semiconductors.

## 2. Results and Discussion

### 2.1. Material Synthesis and Characterizations

**Scheme 1:**
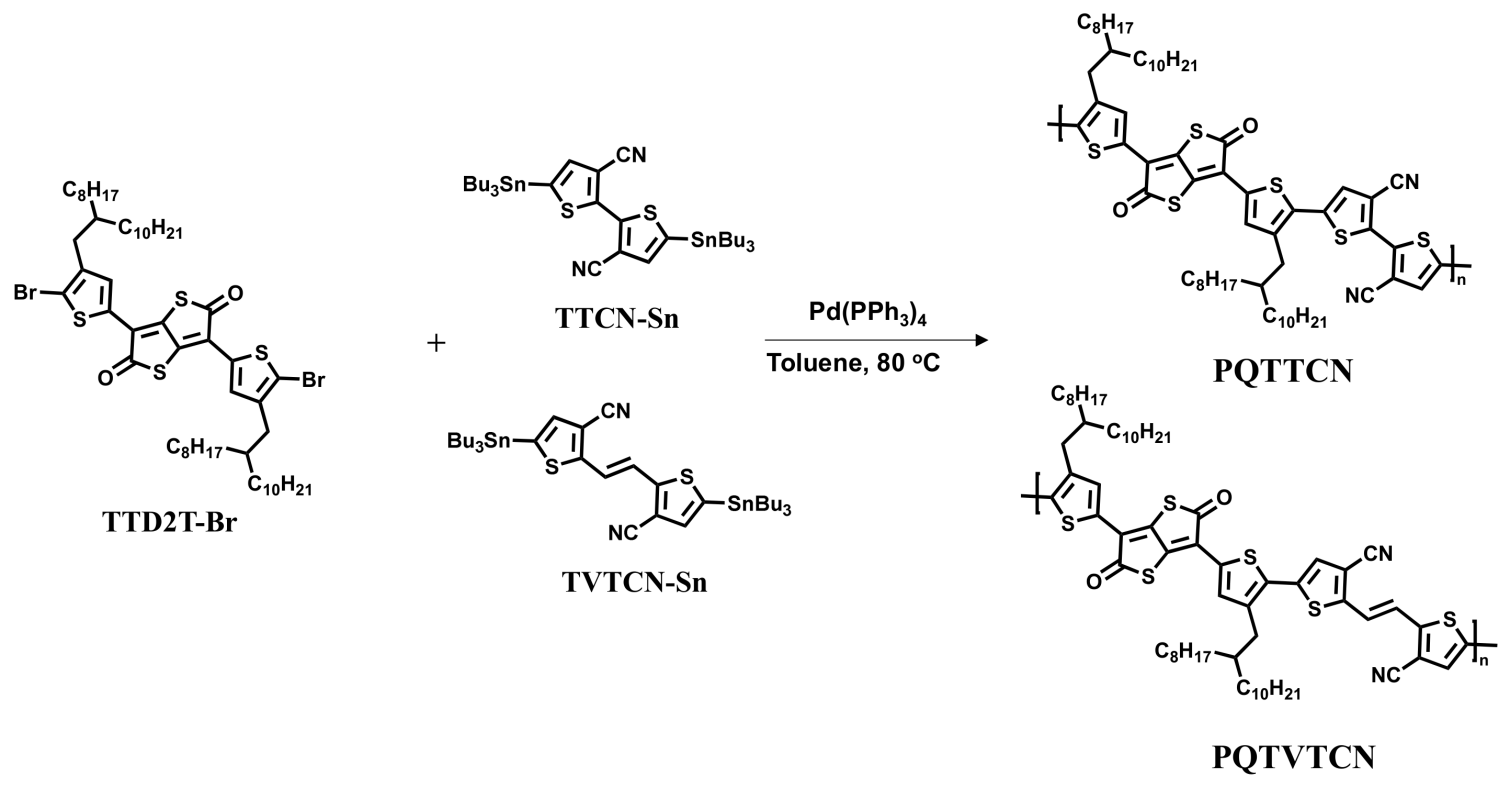
Synthetic routes to polymers PQTTCN and PQTTVTCN.

The dibrominated thiophene-flanked TTD molecule TTD2T-Br, the distannyl monomers of cyano-functionalized bithiophene (TTCN-Sn) and thienylene-vinylene-thienylene (TVTCN-Sn) were synthesized according to previously reported methods [13,37,38,39]. Subsequently, PQTTCN and PQTVTCN were prepared by the Stille coupling polymerization of TTD2T-Br with TTCN-Sn and TVTCN-Sn, respectively, using Pd(PPh3)4 as a catalyst. The synthesis routes are illustrated in Scheme 1 (see the Supporting Information for synthetic details). After polymerization, the polymers were purified by sequential Soxhlet extraction using methanol, acetone, hexane, and dichloromethane to remove oligomers and impurities. The final fractions collected in chloroform were then concentrated and reprecipitated into methanol to afford the target polymers for material characterization and device fabrication. The number-average molecular weights (*M*_n_) and polydispersity indices (PDIs) of the polymers were determined by high-temperature gel permeation chromatography (GPC) at 150 °C using 1,2,4-trichlorobenzene as the eluent (Figure S1). The resulting values were 14.0 kDa/1.72 for PQTTCN and 6.6 kDa/2.03 for PQTVTCN. The relatively low *M*_n_ of PQTVTCN likely stems from solubility limitations caused by its longer conjugated length relative to that of PQTTCN. It is worth noting that both polymers are soluble in chloroform at concentrations of at least 5 mg/mL, which is sufficient for solution processing (e.g., spin coating) for device fabrication, as well as for GPC and ^1^H NMR measurements. Thermogravimetric analysis (TGA) and differential scanning calorimetry (DSC) were conducted to investigate the thermal properties of the two polymers (Figure S2). Both polymers exhibited good thermal stability, with decomposition temperatures (defined as the temperature at 5% weight loss) exceeding 380 °C. For both polymers, no observable phase transition peaks were detected in the temperature window from 30 to 300 °C, which points to a low crystallinity of PQTTCN and PQTVTCN.

### 2.2. Electrochemical and Optical Properties

Ultraviolet-visible-near-infrared (UV-vis-NIR) absorption spectroscopy was employed to investigate the optical properties of PQTTCN and PQTVTCN in both dilute chloroform solutions and thin films, and the corresponding data are listed in Table 1. In dilute solution, both PQTTCN and PQTVTCN exhibit broad absorption bands with absorption maxima (*λ*_max_) at around 640 nm. In thin films, the *λ*_max_^film^ values are red-shifted to 695 nm for PQTTCN and to 708 nm for PQTVTCN, indicating the compact intermolecular packing, likely attributable to the planar and rigid backbones of these two conjugated polymers. Based on the onsets of the thin-film spectra, their optical bandgaps (*E*_g_^opt^) were estimated to be 1.41 and 1.36 eV for PQTTCN and PQTVTCN, respectively. The smaller *E*_g_^opt^ of PQTVTCN versus PQTTCN is in line with the trend observed for the electrochemically determined *E*_g_^CV^ values of the two polymers, as shown below.

**Figure 2:**
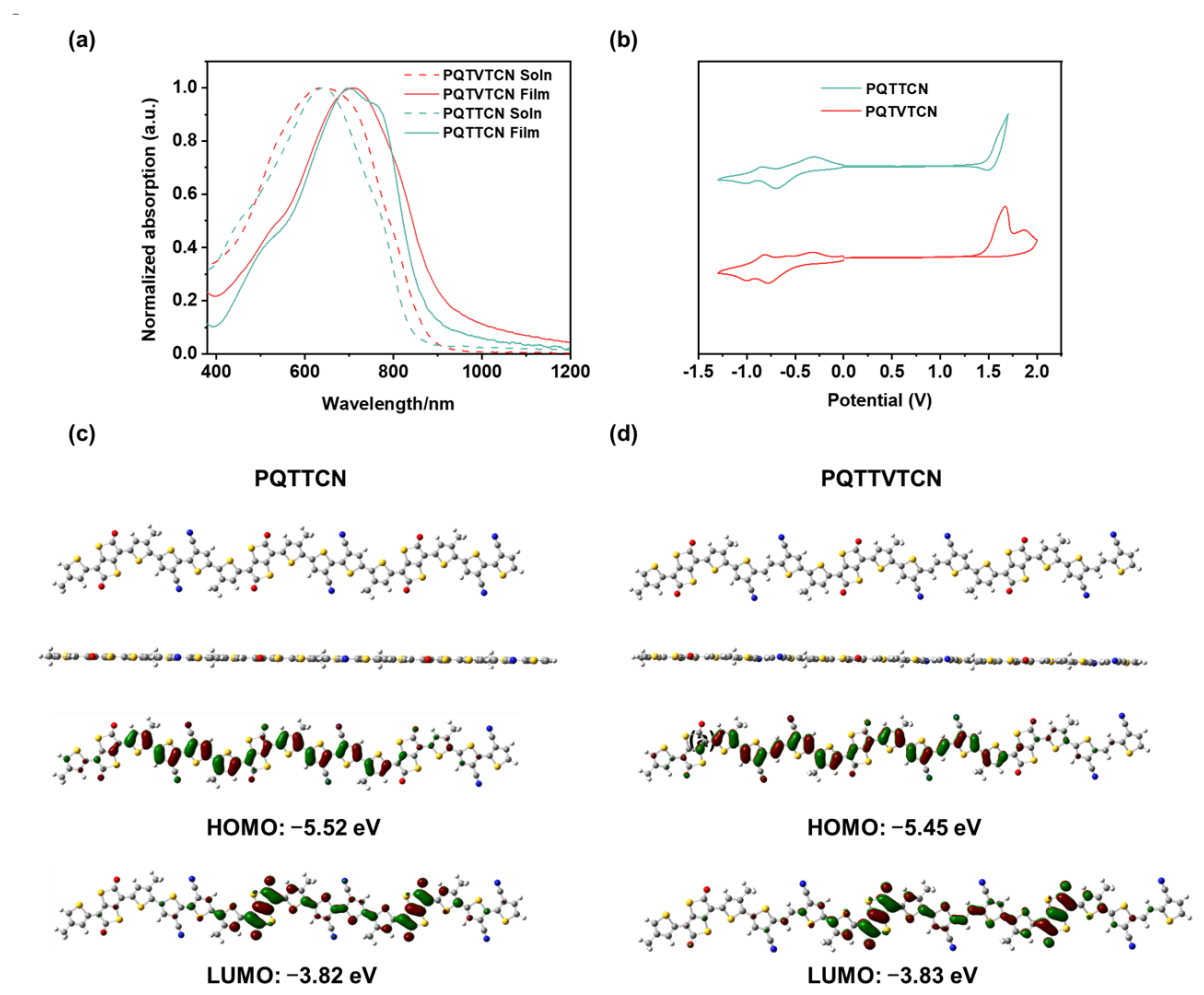
(**a**) Normalized UV-vis-NIR absorption spectra of two conjugated polymers in chloroform solutions (dashed line) and film states (solid line); (**b**) cyclic voltammograms of PQTTCN and PQTVTCN measured in 0.1 M tetrabutylammonium hexafluorophosphate acetonitrile solutions with the Fc/Fc^+^ redox couple as the external standard. (**c**,**d**) Optimized molecular geometries (at the B3LYP/6-31G** level) and HOMO/LUMO topologies for the trimeric models of PQTTCN (**left**) and PQTVTCN (**right**). The alkyl side chains were replaced with methyl groups to simplify the calculations.

Cyclic voltammogram (CV) was employed to investigate the HOMO and LUMO levels (*E*_HOMO_ and *E*_LUMO_) of the two polymers, which were prepared as drop-cast thin films on a glassy carbon electrode, as shown in Figure 2b. The CV curves exhibit both reduction and oxidation peaks for PQTVTCN and PQTTCN. The *E*_HOMO_ and *E*_LUMO_ values calculated from the onset potentials of the reduction and oxidation peaks were determined to be −5.91/−4.07 eV for PQTTCN and −5.87/−4.07 eV for PQTVTCN. The *E*_LUMO_ values are lower than those of many typical n-type polymers, such as the naphthalene diimide-based polymer N2200 (−3.9 eV) [40], the imide-based polymer PCNI-BTI (−3.61 eV) [41], the pyrazine-flanked diketopyrrolopyrrole-based polymer P(PzDPP-CT2) (−4.03 eV) [31], and the B←N bridged bipyridine-based polymer PBN-27 (−3.82 eV) [42]. Additionally, the deep-lying FMOs are expected to facilitate electron injection, suppress hole accumulation, and enhance n-doping efficiency, thereby leading to unipolar n-type characteristics in OFET and OTE devices.

### 2.3. Molecular Structure Analysis

To further investigate the effects of quinoid and cyano groups on the structural and electronic properties of the two polymers, density functional theory (DFT) calculations were conducted at the B3LYP/6-31G** level. As shown in Figure 2c,d, the DFT-optimized structures reveal highly planar backbones for both polymers. Such planarity facilitates intermolecular stacking and is conducive to efficient charge transport. Importantly, these results demonstrate that the introduction of cyano and quinoid groups does not adversely affect the planarity of the polymer backbones. Regarding the calculated HOMO and LUMO topologies, the HOMO wavefunctions of both PQTTCN and PQTVTCN are delocalized across the entire polymer backbones. In contrast, the LUMO wavefunctions are predominantly confined to the electron-deficient TTD moiety. Notably, both polymers exhibit lower LUMO/HOMO levels than polythiophene, with calculated values of −3.82/−5.52 eV for PQTTCN and −3.83/−5.45 eV for PQTVTCN. These results suggest that incorporating both quinoid and cyano groups into the polymer backbone holds great promise for the development of high-performance n-type polymer semiconductors.

### 2.4. Charge Transport and Thermoelectric Performance

**Figure 3:**
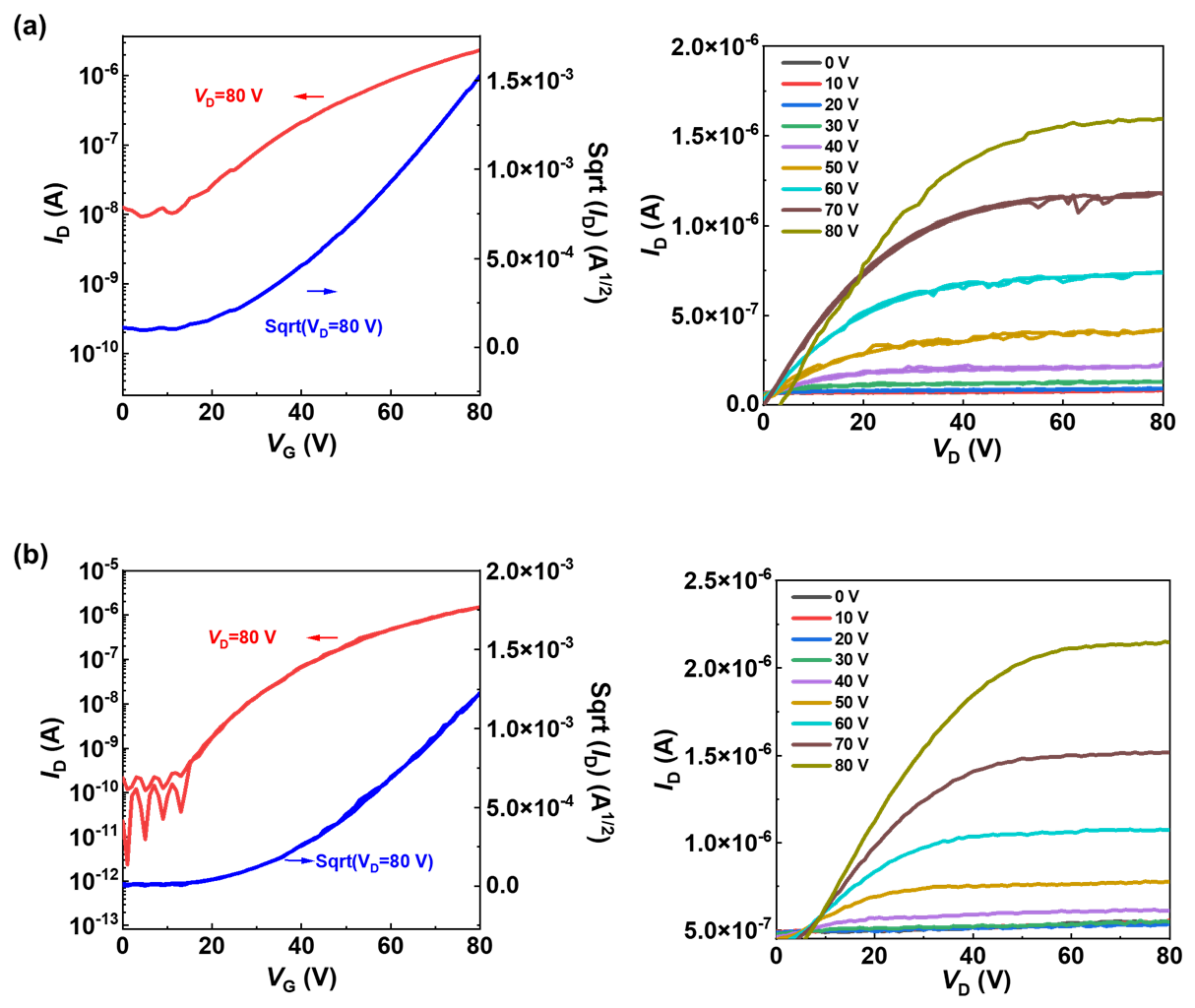
Transfer and output curves of the OFET device based on (**a**) PQTVTCN and (**b**) PQTTCN.

To explore the charge transport properties of the polymers, OFET devices were fabricated using a top-gate/bottom-contact (TGBC) architecture of glass/Au/polymer semiconductor/poly(perfluorobutenylvinylether) (CYTOP)/Al. Source/drain (S/D) electrodes were patterned on glass substrates using photolithography to fabricate devices with a fixed channel width (*W*) of 5 mm and various channel lengths (*L*) of 10, 20, 50, and 100 μm. The active layers were then deposited by spin coating from a 5 mg/mL solution in chloroform. The transfer and output characteristics of PQTTCN and PQTVTCN are displayed in Figure 3a, and the corresponding performance parameters are summarized in Table S1. Both polymers exhibit pronounced unipolar n-type transistor characteristics, attributed to their low LUMO/HOMO levels of PQTVTCN and PQTTCN, which facilitate electron injection and suppress hole injection. This behavior is corroborated by the observed increase in *I*_D_ as the positive *V*_G_ increases. The device parameters were obtained from the transfer curves in the saturation regime, yielding for PQTTCN a *μ*_e_ of 0.036 cm^2^ V^−1^ s^−1^, a threshold voltage (*V*_th_) of 32.76 V, and an on/off current ratio (*I*_on_/*I*_off_) of 10^4^; for PQTVTCN, the corresponding values were 0.002 cm^2^ V^−1^ s^−1^, 37.32 V, and 10^2^, respectively. The increased *µ*_e_ of PQTTCN is mainly due to its higher *M*_n_ (14 kDa) than that of PQTVTCN (6.6 kDa), which can facilitate intramolecular charge delocalization and interchain hopping. In contrast to other polythiophene derivatives, which typically exhibit p-type behavior, PQTTCN and PQTVTCN show exclusively unipolar n-type charge transport in OFETs. This distinct behavior demonstrates that the dual-functionalization strategy with quinoid and cyano groups provides a powerful approach for constructing unipolar n-type polymer semiconductors.

**Figure 4:**
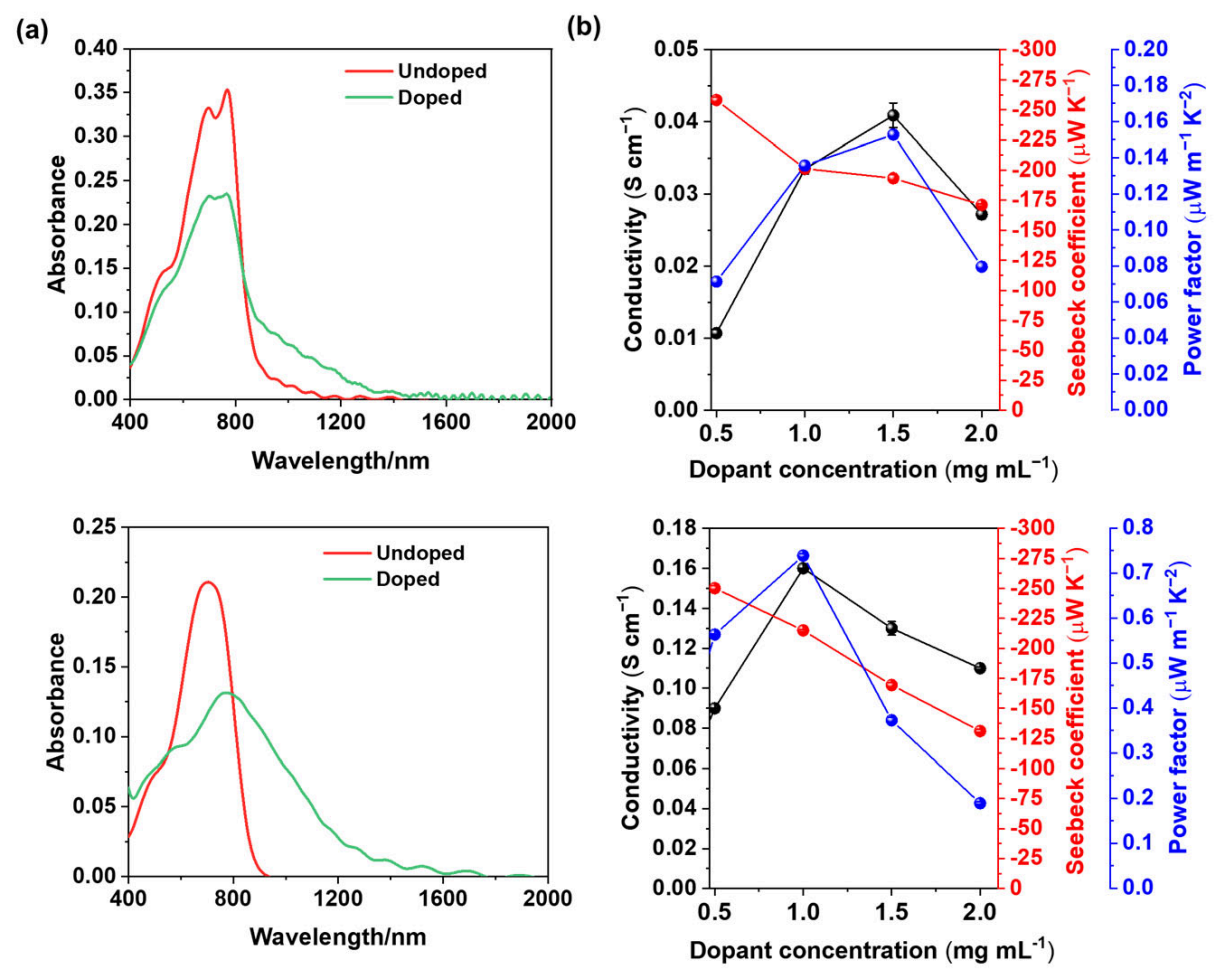
(**a**) UV-vis-NIR spectra of PQTVTCN (**up**) and PQTTCN (**bottom**) films before and after doping. (**b**) Average σ, S, and PF values measured for doped PQTVTCN (**up**) and PQTTCN (**bottom**) films as a function of JLBI dopant concentration.

The deep-lying LUMO levels of PQTTCN and PQTVTCN indicate that these polymers can be readily n-doped. Given this characteristic, the n-dopant JLBI was selected due to its strong n-doping ability and good solution processability [43,44]. In addition, JLBI exhibits significantly higher lipophilicity than typical dopants such as *N*, *N*-dimethyl-2-phenyl-2,3-dihydro-1*H*-benzoimidazole (N-DMBI) or trisaminomethane derivative (TAM) due to its two fused cycloalkyl edges, which promote affinity toward alkylated n-type organic semiconductors. To evaluate the n-doping efficiency, UV-vis-NIR absorption measurements were conducted. Both polymers were subjected to sequential n-doping via transition metal-catalyzed doping technique [45]. Upon doping, blending the pristine polymer films with JLBI led to progressive bleaching of the 500–900 nm absorption band (Figure 4a), accompanied by the emergence of a broad polaronic band in the near-infrared region (>1000 nm), indicative of efficient doping. Notably, PQTTCN shows a stronger polaron absorption than PQTVTCN, suggesting that the former can be more readily doped than the latter. This result was further supported by electron paramagnetic resonance (EPR) measurements, as shown in Figure S3. A higher intensity of radical signal was observed for PQTTCN than PQTVTCN, suggesting an increased charge concentration of the doped PQTTCN film, which should contribute to a higher σ of this polymer.

The thermoelectric efficiency of OTE devices is typically quantified by the dimensionless figure of merit, *ZT* = *S*^2^σT/*κ*, where *S*, σ, T, and *κ* are the Seebeck coefficient, electrical conductivity, absolute temperature, and thermal conductivity, respectively [46]. However, because the κ of organic materials is intrinsically low and challenging to measure precisely, the power factor (PF = *S*^2^σ) is commonly used as a practical alternative for evaluating OTE performance [47,48]. Thermoelectric devices were fabricated on borosilicate glass substrates. The substrates were first placed in a N2-filled glove box, where Au electrodes (thickness: 30 nm; length: 100 μm; width: 2000 μm) were thermally evaporated through a shadow mask. After that, a 0.1 nm gold nanoparticle (Au-NP) layer was deposited by thermal evaporation onto the substrates. Next, PQTTCN and PQTVTCN polymer solutions in chloroform (5 mg/mL) were spin-coated at 1000 rpm for 45 s and then annealed at 240 °C for 15 min. Finally, a sequential doping process was applied, in which JLBI dopant solutions (0.5–2 mg/mL in n-butyl acetate) were spin-coated over the semiconducting layer at 1500 rpm for 30 s, followed by a brief annealing at 120 °C for 15 s to complete the doping activation.

To assess the thermoelectric performance of the doped polymers, we measured their σ and *S* as functions of the JLBI concentration, which was varied from 0.5 to 2.0 mg mL^−1^ in increments of 0.5 mg mL^−1^ (Figure 4a and Table 2). The maximum σ values of 0.04 S cm^−1^ for PQTVTCN and 0.16 S cm^−1^ for PQTTCN were achieved at dopant concentrations of 1.5 and 1.0 mg mL^−1^, respectively. The markedly higher σ of PQTTCN relative to PQTVTCN can be ascribed to superior *μ*_e_ and stronger n-doping efficiency for the former, as corroborated by OFET and absorption spectroscopy measurements. Beyond these optimal concentrations, further increasing the dopant loading led to a gradual decline in σ, likely due to carrier–carrier repulsions and morphological disruption in the heavily doped state [49,50].

The *S* values of the JLBI-doped PQTTCN and PQTVTCN films were subsequently determined by measuring the thermovoltages generated upon applying distinct temperature gradients across the samples from 0 to 2.8 K (in 0.7 K steps; Figures S4 and S5). Notably, all the doped polymer films exhibit negative *S* values, confirming n-type doping. Moreover, the absolute *S* value decreased progressively with increasing dopant concentration (0.5–2.0 mg mL^−1^), consistent with the well-known inverse relationship between *S* and charge carrier concentration. By optimizing the trade-off between σ and *S*, the highest power factors were obtained at JLBI concentrations of 1.5 mg mL^−1^ for PQTVTCN and 1.0 mg mL^−1^ for PQTTCN, yielding PF values of 0.17 and 0.75 μW m^−1^ K^−2^, respectively. The superior thermoelectric performance of PQTTCN over PQTVTCN can be attributed to its higher *μ*_e_ and n-doping efficiency, as demonstrated by OFET measurements and UV-vis-NIR absorption spectroscopy, respectively. Collectively, these findings confirm the incorporation of cyano and quinoid groups into the polymer backbone as an effective strategy for advancing n-type OTE applications.

### 2.5. Film Microstructures and Morphology

**Figure 5:**
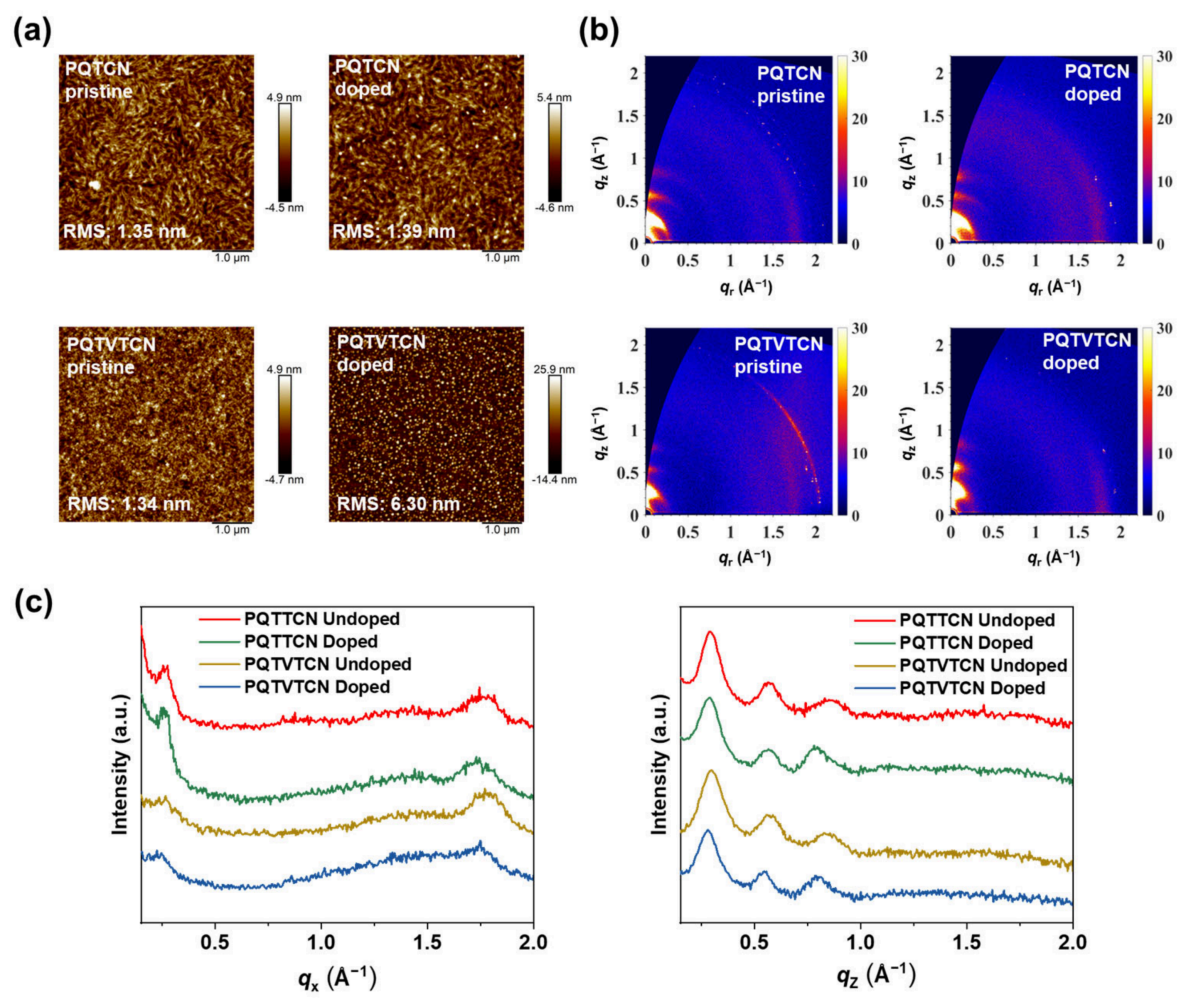
(**a**) AFM images and (**b**) GIWAXS images of PQTTCN and PQTVTCN films before and after doping by JLBI; (**c**) IP and OOP line-cut profiles of the GIWAXS images.

Atomic force microscopy (AFM) was employed to investigate the surface morphology of the polymer films. As shown in Figure 5a, the pristine films of PQTTCN and PQTVTCN displayed smooth and uniform surfaces with a small root-mean-square (RMS) roughness value of approximately 1.3 nm, indicating low surface roughness for both polymers. Upon doping, however, the two polymers display markedly different morphological evolution. The RMS roughness of PQTTCN increase only slightly from 1.32 to 1.39 nm, whereas that of PQTVTCN undergoes a pronounced increase from 1.34 to 6.30 nm. This marked contrast suggests that PQTTCN possesses better compatibility with the dopant than PQTVTCN, which thereby achieves a higher σ.

Grazing-incidence wide-angle X-ray scattering (GIWAXS) measurements were also carried out to gain deeper insights into the polymer chain packing of PQTTCN and PQTVTCN films before and after doping, and the corresponding GIWAXS images and one-dimensional line profiles are presented in Figure 5b,c. In the in-plane (IP) direction, the pristine films of both polymers adopt an edge-on orientation with prominent (010) diffraction peaks at ∼1.77 Å^−1^, corresponding to a close π-π stacking distance of ∼3.55 Å. Additionally, a stronger (100) diffraction peak of PQTTCN is observed relative to PQTVTCN, indicative of enhanced crystallinity in the former. This observation is quantitatively supported by the crystalline coherence lengths (CCL) for the (100) peaks (70.0 Å for PQTTCN versus 60.2 Å for PQTVTCN), confirming the superior crystallinity of PQTTCN, which likely contributes to its enhanced *μ*_e_. Upon doping, however, no significant changes in the diffraction peaks are observed for either polymer, suggesting that the crystalline microstructure is largely preserved after the doping process. In the out-of-plane direction, the diffraction peaks of both pristine and doped films remain nearly identical for the two polymers, which is probably ascribed to their structurally similar backbones. Taken together, the GIWAXS results indicate that the primary factor governing the performance difference between the two polymers lies in their crystallinity in the IP direction.

## 3. Conclusions

In summary, we have successfully developed a dual-functionalization strategy for constructing n-type polythiophene derivatives by integrating both quinoid and cyano groups into the polymer backbone, yielding PQTTCN and PQTVTCN. Benefiting from the strong electron-withdrawing nature of the quinoid core and the cyano substituents, both polymers exhibit deep-lying LUMO and HOMO levels (below −4.0 and −5.8 eV, respectively) and highly planar backbones, as corroborated by electrochemical, optical and DFT studies. Consequently, PQTTCN and PQTVTCN show unipolar n-type charge transport in OFETs, with μe of 0.036 and 0.002 cm^2^ V^−1^ s^−1^, respectively, representing rare examples of n-type behavior in polythiophene-based systems. Furthermore, after n-doping with JLBI, both polymers exhibit n-type thermoelectric performance, with σ and PF values of 0.16 S cm^−1^ and 0.75 μW m^−1^ K^−2^ for PQTTCN and 0.043 S cm^−1^ and 0.17 μW m^−1^ K^−2^ for PQTVTCN, respectively. AFM and GIWAXS analyses reveal that the superior performance of PQTTCN stems from its better dopant compatibility and its higher crystallinity along the in-plane direction. Meanwhile, both polymers retain their crystalline microstructures after doping, with no significant diffraction peak shifts, indicating robust film microstructures. These results demonstrate that the synergistic incorporation of thienoquinoid and cyano groups effectively lowers FMO levels, thereby suppressing hole transport and enabling unipolar n-type performance in polythiophene derivatives. This dual-functionalization strategy therefore provides a promising route for the development of high-performance n-type polymer semiconductors.

## Figures and Tables

**Table 1:** Summary of molecular weights, optical bandgaps, and electrochemical properties of PQTTCN and PQTVTCN.

Polymer	*M*_n_ ^1^ (kDa)	PDI ^1^	*λ*_max_ ^2^ (nm)	*E*_g_^opt 3^ (eV)	*E*_HOMO_ ^4^ (eV)	*E*_LUMO_ ^4^ (eV)
PQTVTCN	6.6	2.03	708	1.36	−5.81	−4.07
PQTTCN	14	1.72	695	1.41	−5.91	−4.07

^1^ Measured by GPC using trichlorobenzene as eluent at 150 °C. ^2^ Determined from thin-film absorption spectra. ^3^ Estimated from the absorption onset of as-cast films using the equation: *E*_g_^opt^ = 1240/*λ*_onset_. ^4^ Calculated using the equations *E*_HOMO_ = −(*E*_ox_ + 4.8) eV and *E*_LUMO_ = −(*E*_red_ + 4.8) eV, where *E*_ox_ and *E*_red_ represent the oxidation and reduction onset potentials (vs. Fc/Fc^+^), respectively.

**Table 2:** Maximum and average (in parenthesis) OTE performance parameters of PQTTCN and PQTVTCN after doping with JLBI. The average values are derived from data collected from at least five devices.

Polymer	σ (S cm^−1^)	*S* (μV K^−1^)	PF (μW m^−1^ K^−2^)
PQTVTCN	0.043 (0.041 ± 1.1 × 10^−3^)	−197.01 (−193.25 ± 2.92)	0.17 (0.15 ± 0.01)
PQTTCN	0.160 (0.155 ± 1.2 × 10^−4^)	−216.12 (−214.9 ± 0.96)	0.75 (0.74 ± 0.004)

## Data Availability

All data supporting the findings of this study are included within the article.

## Supplementary Materials

The following supporting information can be downloaded at: https://www.mdpi.com/article/10.3390/polym18151900/s1, Figure S1: GPC curves of PQTTCN and PQTVTCN; Table S1: Charge transport properties of polymers evaluated as OFETs; Figure S2: TGA (a) and DSC (b) curves of PQTTCN and PQTVTCN; Figure S3: EPR signals of PQTTCN and PQTVTCN after n-doping with JLBI; Figure S4: Representative thermal voltage response curves for sequentially doped PQTTCN polymer films at varying JLBI concentrations: (a) 0.5 mg mL^−1^, (b) 1 mg mL^−1^, (c) 1.5 mg mL^−1^ and (d) 2 mg mL^−1^. Temperature differences ranging from 0 to 2.8 K (by step of 0.7 K) were applied across the doped polymer films, followed by the return to 0 K; Figure S5: Representative thermal voltage response curves for sequentially doped PQTVTCN polymer films at varying JLBI concentrations: (a) 0.5 mg mL^−1^, (b) 1 mg mL^−1^, (c) 1.5 mg mL^−1^ and (d) 2 mg mL^−1^. Temperature differences ranging from 0 to 2.8 K (by step of 0.7 K) were applied across the doped polymer films, followed by the return to 0 K; Figure S6: ^1^H NMR spectrum of TTCN-Sn; Figure S7: ^13^C NMR spectrum of TTCN-Sn; Figure S8: ^1^H NMR spectrum of TVTCN-Sn; Figure S9: ^13^C NMR spectrum of TVTCN-Sn; Figure S10: ^1^H NMR spectrum of TTD2T-Br; Figure S11: ^13^C NMR spectrum of TTD2T-Br; Figure S12: ^1^H NMR spectrum of PQTTCN; Figure S13: ^1^H NMR spectrum of PQTVTCN.
