# Supplementary material for: Backbone Engineering of Polythiophenes via Quinoid and Cyano Dual Functionalization for n-Type Polymers
Source: Polymers (Basel). 2026 Aug 3;18(15):1900. doi: 10.3390/polym18151900 (PMC13468699; doi:10.3390/polym18151900)
Supplement: Supplementary file 1 [file polymers-18-01900-s001.zip › polymers-4448541-supplementary.pdf]

Backbone Engineering of Polythiophenes via Quinoid and Cyano Dual Functionalization for n-Type Polymers

Weipeng Sun [1, †, *], Yanlin Wei [2, †], Peng Wang [2], Dingqin Hu [2], Peng Dai [1], Wenge Zhang [1], Dian Zhang [5], Jianfeng Li [4], Yongqiang Shi [3], Xugang Guo [2, *]

[1] Anhui Provincial Key Laboratory of Advanced Catalysis and Energy Materials, Anhui Key Laboratory of Optoelectronic Magnetic Functional Complex and Nano Complex, School of Chemistry and Chemical Engineering, Anqing Normal University, Anqing 246133, China

[2] Shenzhen Key Laboratory of Printed Electronics, Department of Materials Science and Engineering, Southern University of Science and Technology, Shenzhen, Guangdong 518055, China

[3] Key Laboratory of Functional Molecular Solids, Ministry of Education, School of Chemistry and Materials Science, Anhui Normal University, No.189, Jiuhua South Road, Wuhu, Anhui, 241002, China

[4] School of Materials Science and Engineering, Shaanxi Normal University, Xi'an, Shaanxi 710119, China

[5] Institute of Industrial Science, the University of Tokyo, Komaba 4-6-1, Meguro-ku, Tokyo 153-8505, Japan

* Correspondence: sunwp@aqnu.edu.cn (W.S); guoxg@sustech.edu.cn (X.G)

† These authors contributed equally to this work.

Content

S1. Materials..3

S2. Measurements and instrumentations...3

S3. Synthesis Procedures...4

S4. Polymer thermal properties..5

S5. OTE devices characterization...6

S6. OTE devices characterization...9

S7. References...13

S1. Materials

All reagents and chemicals were purchased from Energy Chemical, and Bide Pharmatch Ltd., *etc*, and were used as received without further purification unless otherwise specified. TTD2T-Br and TTCN-Sn and TVTCN-Sn were synthesized according to previously reported procedures.[1-3] Anhydrous toluene was distilled from Na/benzophenone under an argon atmosphere. Unless otherwise stated, all reactions were carried out under an inert atmosphere using standard Schlenk line techniques.

S2. Measurements and instrumentations

Polymer molecular weights were determined using a Polymer Laboratories GPC-PL220 at 40 versus polystyrene standards with 1,2,4-trichlorobenzene as the eluent. Cyclic voltammetry (CV) measurements of polymer films were conducted on a CHI660 potentiostat/galvanostat electrochemical workstation, employing a platinum disk as the working electrode, a platinum wire as the counter electrode, and an Ag/AgCl reference electrode in a 0.1 M tetrabutylammonium hexafluorophosphate acetonitrile solution as the supporting electrolyte. The lowest unoccupied molecular orbital (LUMO) and the highest occupied molecular orbital (HOMO) energy levels of the polymers were calculated through the formulas: $E_{\text{LUMO/HOMO}} = -(4.80+E_{\text{onset}}^{\text{red}}/E_{\text{onset}}^{\text{ox}})$ eV. UV-Vis-NIR absorption spectra of the polymer in solution state were collected on a Shimadzu UV-3600 UV-Vis-NIR spectrophotometer. Thermogravimetric analysis (TGA) was conducted on the same instrument. Differential scanning calorimetry (DSC) curves were recorded on a Mettler STARe system (TA Instrument) under nitrogen with a heating ramp of 10 ºC min^{-1}. Grazing-incidence wide-angle X-ray scattering (2D-GIWAXS) measurements were carried out with a Xeuss 2.0 SAXS laboratory beamline using a Cu X-ray source (8.05 keV, 1.54 Å) and a Pilatus3R 300K detector. The incidence angle is 0.2°. Atomic force microscopy (AFM) was employed to characterize the surface morphology of polymer films using a Dimension Icon Scanning Probe Microscope (Asylum Research, MFP-3D-Stand Alone) operated in tapping mode. The configuration optimizations of the polymer were calculated at density functional theory

(DFT) calculations using the Gaussian 09 program at the B3LYP/6-31**. In all cases, the long side chains were replaced with methyl groups to reduce computational cost.

S3. Synthesis Procedures

Synthesis of PQTTCN:

A glass tube was charged with the two monomers, TTCN-Sn (28.5 μmol, 1.0 eq), TTD2T-Br (28.5 μmol, 1.0 eq), and $Pd(PPh_3)_4$ (1.4 μmol, 0.05 eq). The tube and its contents were subjected to three pump-purge cycles with argon, followed by the addition of anhydrous toluene (2.5–3.0 mL) via syringe. The tube was sealed under an argon flow and then stirred under oil bath at 110 °C for 3 h. After cooling to room temperature, the reaction mixture was slowly added dropwise into 50 mL of methanol with vigorous stirring. The resulting mixture was stirred for 30 min, and the solid precipitate was transferred to a Soxhlet thimble. After drying, the crude product was subjected to sequential Soxhlet extraction with methanol, acetone, hexane, dichloromethane, and chloroform. The final chloroform fraction was concentrated by removing most of the solvent, and then the residue was precipitated into methanol. The polymer was collected by filtration, dried under reduced pressure, yielding a deep-colored solid product (yield = 95%). M_n = 14.0 kDa, PDI = 1.72.

Synthesis of PQTVTCN:

A glass tube was charged with the two monomers, TVTCN-Sn (28.5 μmol, 1.0 eq), TTD2T-Br (28.5 μmol, 1.0 eq), and $Pd(PPh_3)_4$ (1.4 μmol, 0.05 eq). The tube and its contents were subjected to three pump-purge cycles with argon, followed by the addition of anhydrous toluene (2.5–3.0 mL) via syringe. The tube was sealed under an argon flow and then stirred under oil bath at 80 °C for 1 h. After cooling to room temperature, the reaction mixture was slowly added dropwise into 50 mL of methanol with vigorous stirring. The resulting mixture was stirred for 30 min, and the solid precipitate was transferred to a Soxhlet thimble. After drying, the crude product was subjected to sequential Soxhlet extraction with methanol, acetone, hexane, dichloromethane, and chloroform. The final chloroform fraction was concentrated by

removing most of the solvent, and then the residue was precipitated into methanol. The polymer was collected by filtration, dried under reduced pressure, yielding a deep-colored solid product (yield = 88%). M_n = 6.6 kDa, PDI = 2.03.

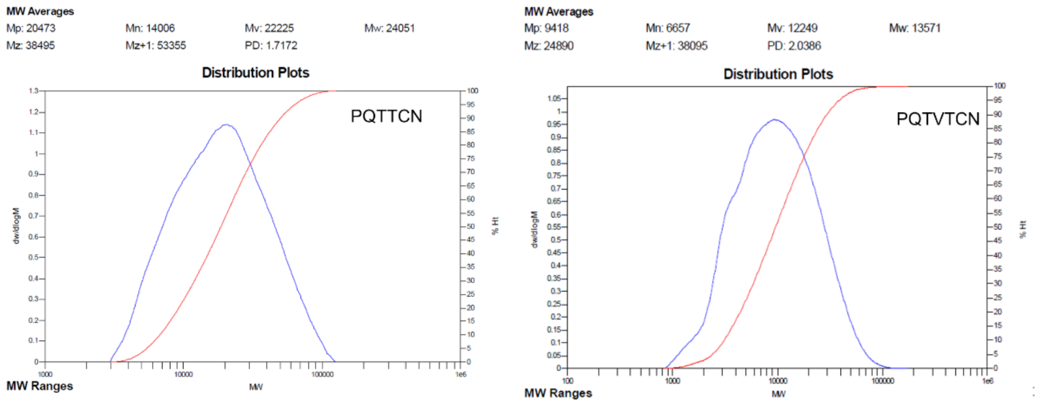

Figure S1. GPC curves of PQTTCN and PQTVTCN.

Table S1: Charge transport properties of polymers evaluated as OFETs

Polymer	μ_e (cm^2 V^{-1} s^{-1}) [a]	V_{th} (V) [b]	I_{on}/I_{off}
PQTTCN	3.62E-2 (±0.005)	32.76 (±1.5)	10^4
PQTVTCN	2.02E-3 (±0.0006)	37.32 (±1.8)	10^2

[a]Average electron mobilities. [b]Average threshold voltages. Parentheses for all the parameters are standard deviations. The average values are derived from data collected from at least five devices.

S4. Polymer thermal properties

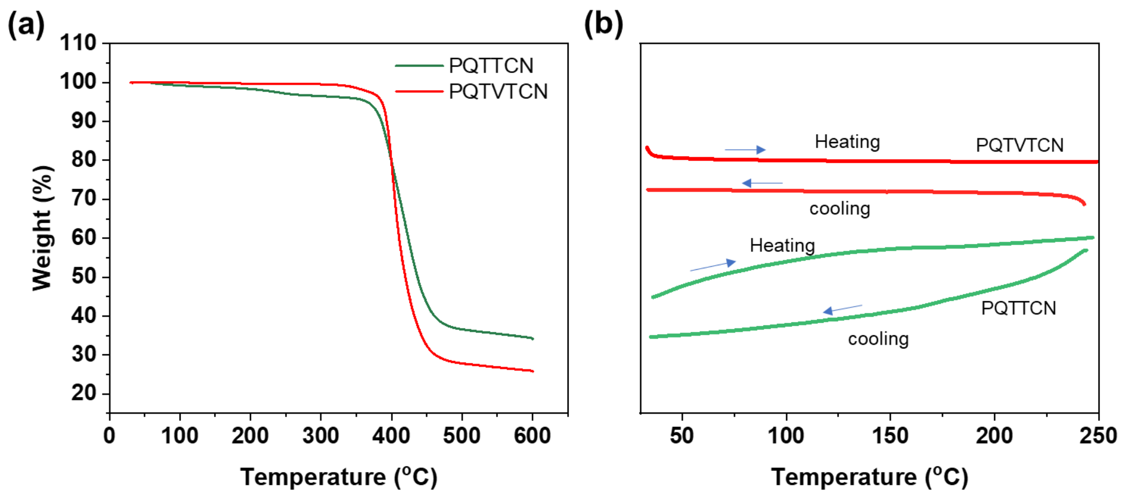

Figure S2. TGA (a) and DSC (b) curves of PQTTCN and PQTVTCN.

S5. OTE devices characterization

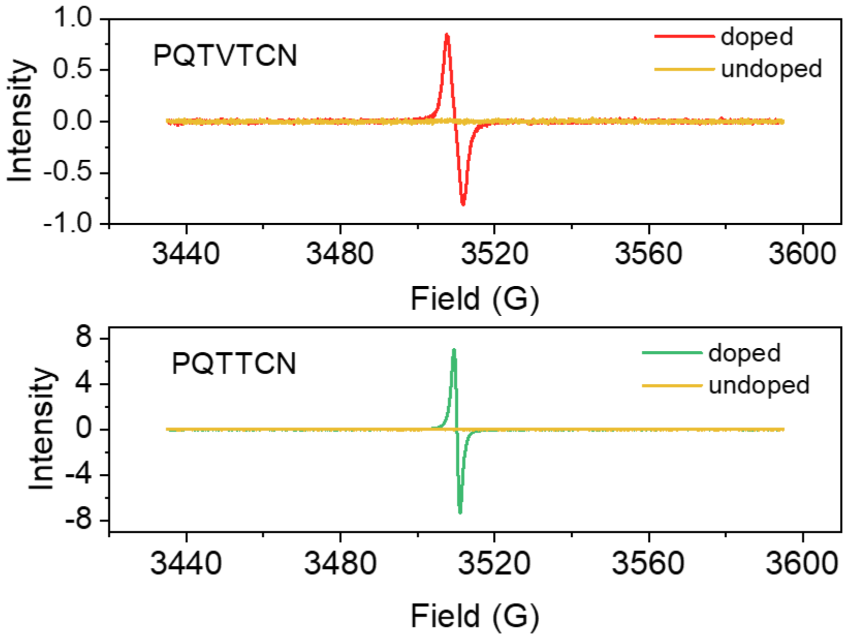

Figure S3. EPR signals of PQTTCN and PQTVTCN after n-doping with JLBI.

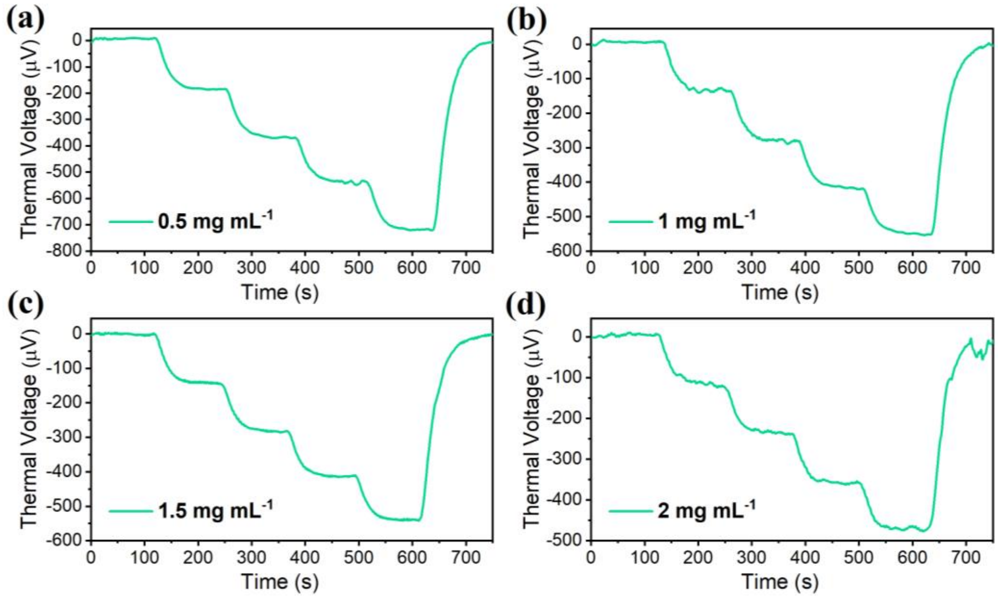

Figure S4. Representative thermal voltage response curves for sequentially doped PQTTCN polymer films at varying JLBI concentrations: (a) 0.5 mg mL^{-1}, (b) 1 mg mL^{-1}, (c) 1.5 mg mL^{-1} and (d) 2 mg mL^{-1}. Temperature differences ranging from 0 to 2.8 K (by step of 0.7 K) were applied across the doped polymer films, followed by the return to 0 K.

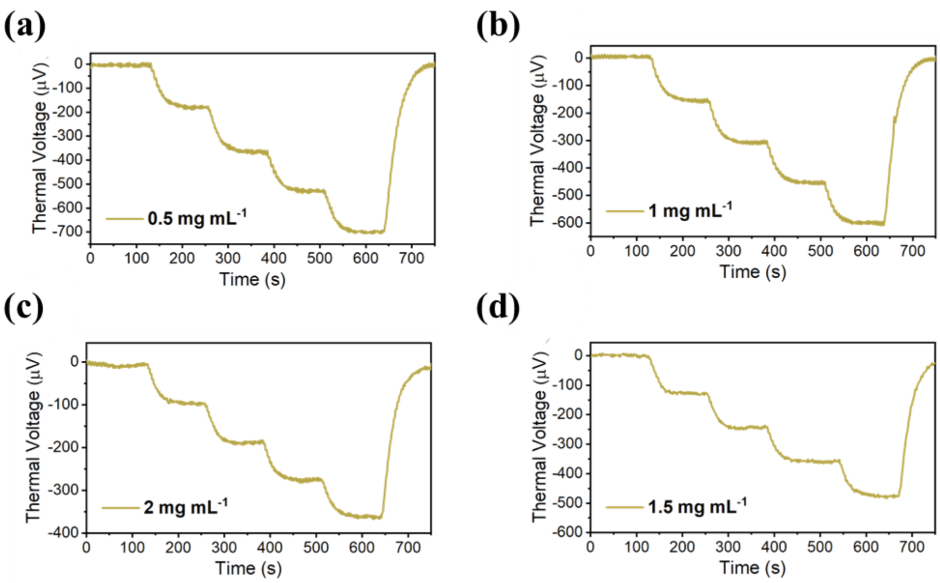

Figure S5. Representative thermal voltage response curves for sequentially doped PQTVTCN polymer films at varying JLBI concentrations: (a) 0.5 mg mL^{-1}, (b) 1 mg mL^{-1}, (c) 1.5 mg mL^{-1} and (d) 2 mg mL^{-1}. Temperature differences ranging from 0 to 2.8 K (by step of 0.7 K) were applied across the doped polymer films, followed by the return to 0 K.

Organic thermoelectric devices (OTEs) were fabricated on Borosilicate glass substrates. First, the substrates were cleaned by sonication washing in isopropanol, acetone and isopropanol successively for 20 min each, followed by 15 min UV-ozone treatment. After that, the substrates were transferred into the N_2-filled glove box, where 30 nm Au electrodes of 100 μm length and 2000 μm width were thermally evaporated through a shadow mask. Once 0.1 nm gold nanoparticles (Au-NPs) were thermally evaporated onto the substrate, 5 mg mL^{-1} polymer solutions in chloroform for PQTTCN and PQTVTCN were spin-coated at 1000 rpm for 45 s and annealed for 15 min at 240 °C. A Doped films were fabricated following a sequential doping procedure, in which JLBI dopant solutions (0.1-2 mg mL^{-1} in *n*-butyl acetate) were spin-coated over the semiconducting layer at 1500 rpm for 30 s, followed by thermal annealing at 120 °C for 15 s to activate the doping. For electrical conductivity measurement, the linear *I-V* curves were characterized in a N_2-filled glove box by using a Keithley 4200-SCS system. Average electrical conductivity values were calculated from five different measurements. On the other hand, thermoelectric performance was measured by using

a pair of Peltier devices with a separation distance of 1 mm, in which temperature differences ranging from 0 to 2.8 K (by step of 0.7 K) were applied between the electrodes by using a pair of thermal couples. In this sense, the thermal voltage measured in a N_2-filled glove box by using a Keithley 4200-SCS system was used to extract Seebeck coefficients through the linear fitting of the thermal voltage versus ΔT. Average Seebeck coefficients were calculated from five different measurements.

S6. NMR

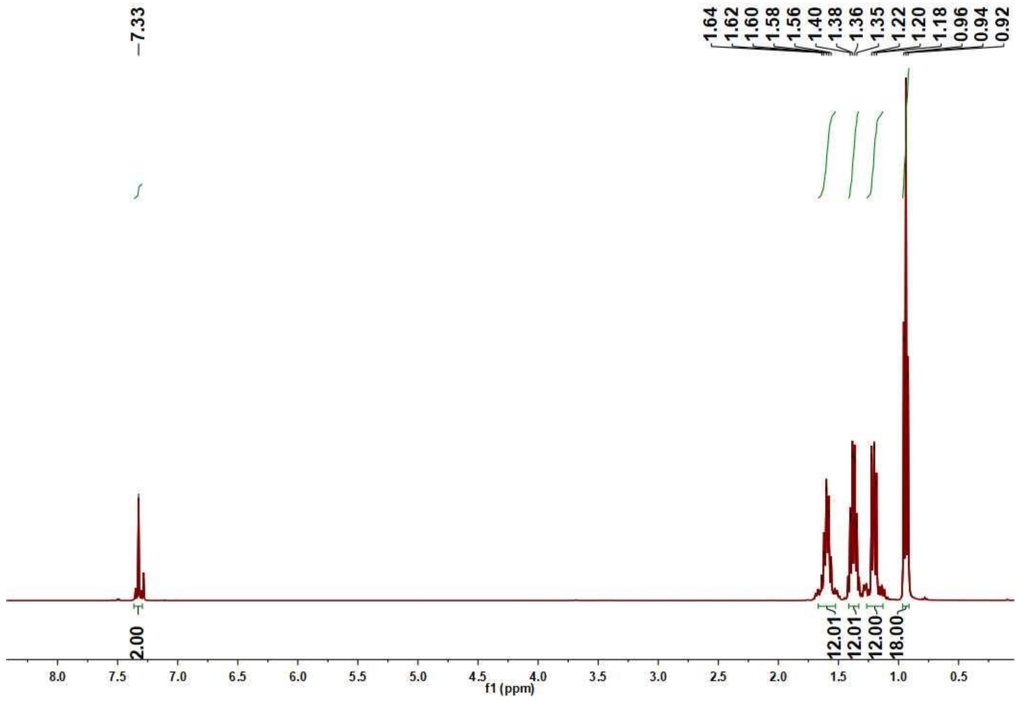

Figure S6. ^{1}H NMR spectrum of TTCN-Sn

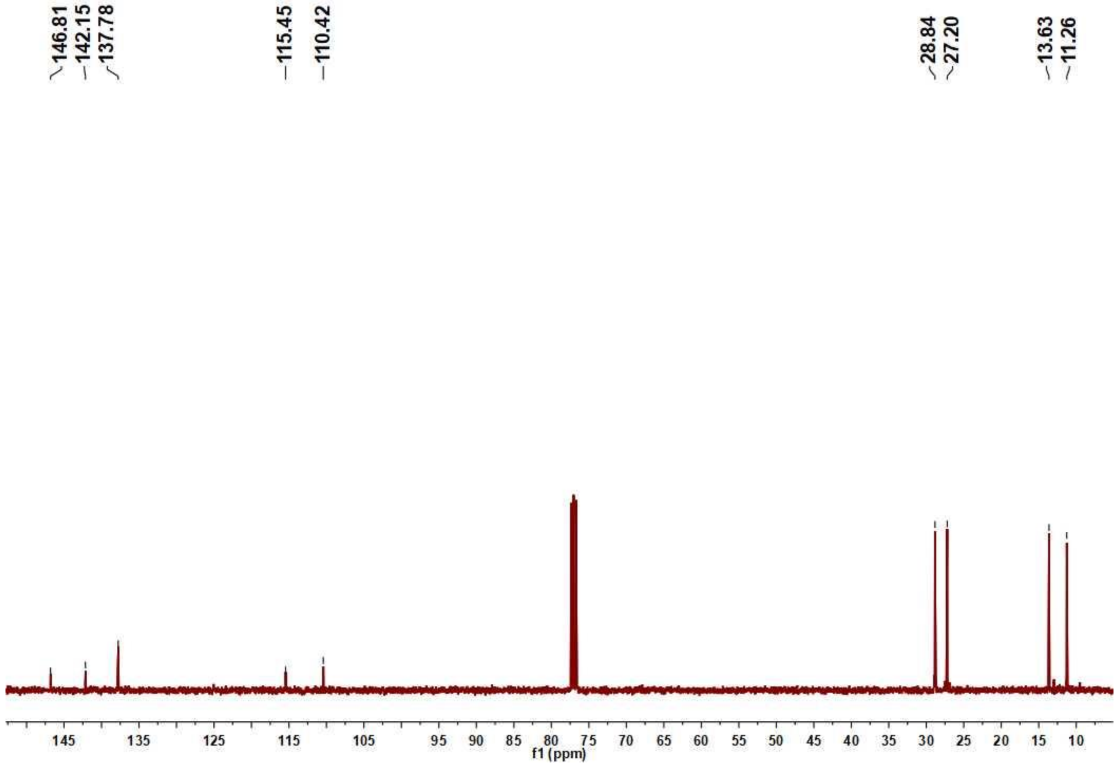

Figure S7. ^{13}C NMR spectrum of TTCN-Sn

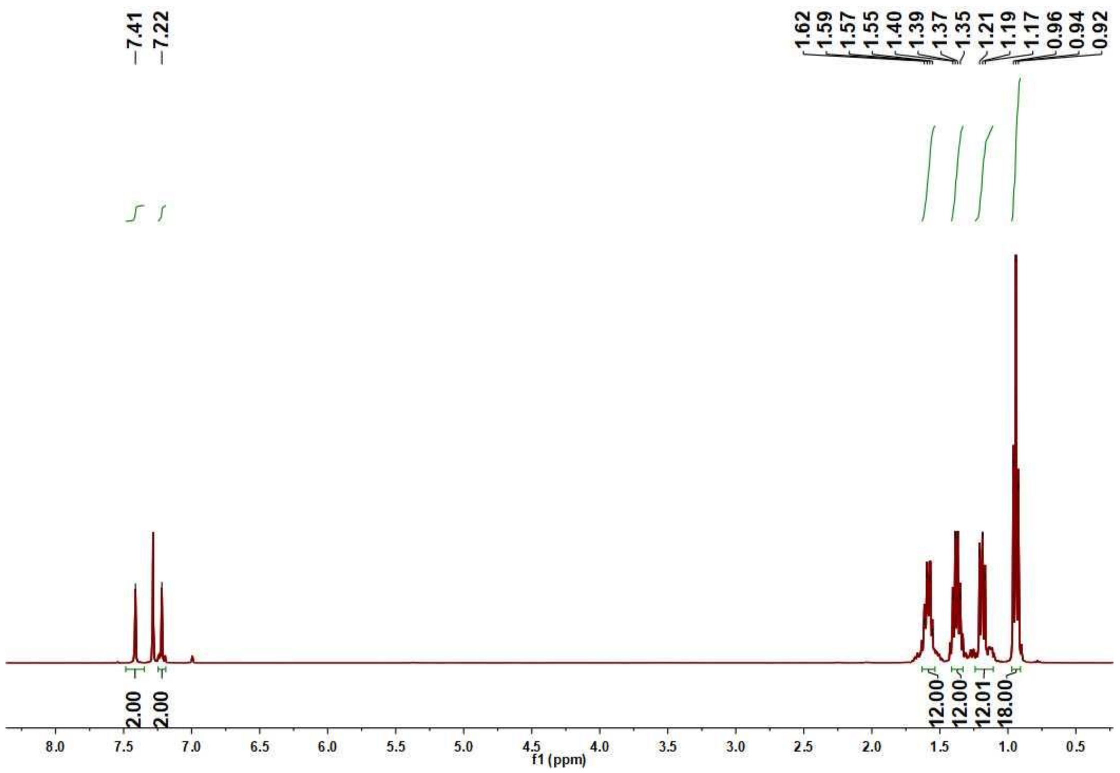

Figure S8. ^{1}H NMR spectrum of TVTCN-Sn

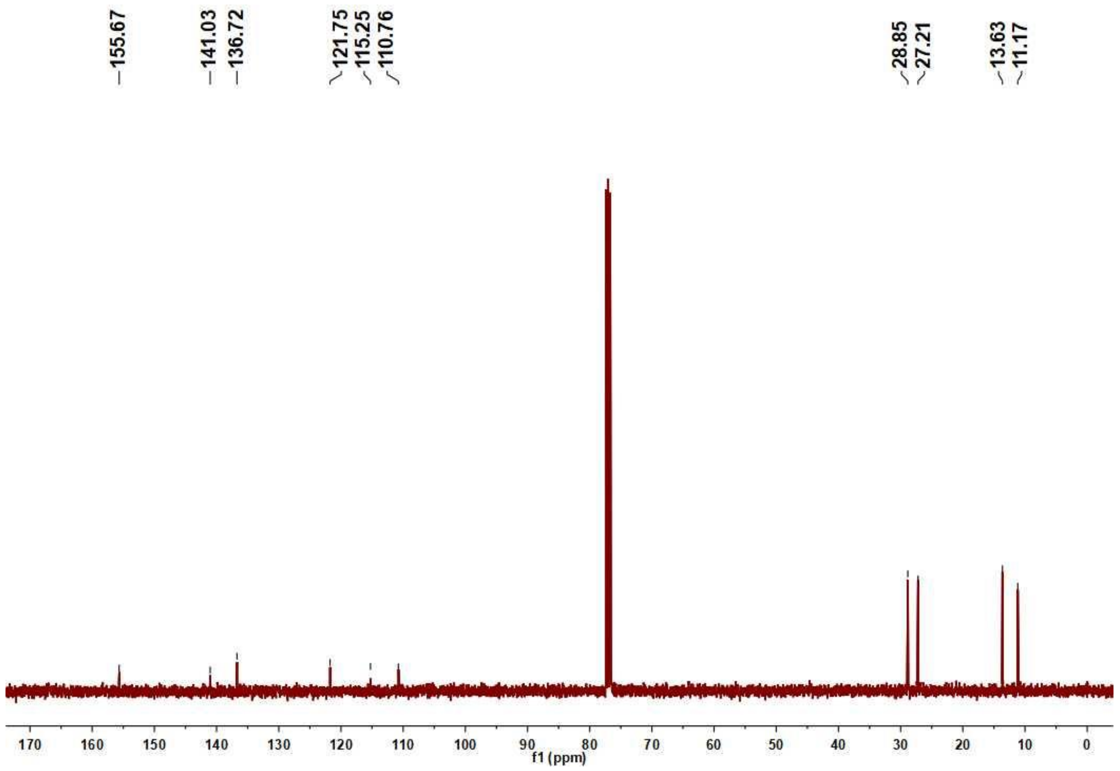

Figure S9. ^{13}C NMR spectrum of TVTCN-Sn

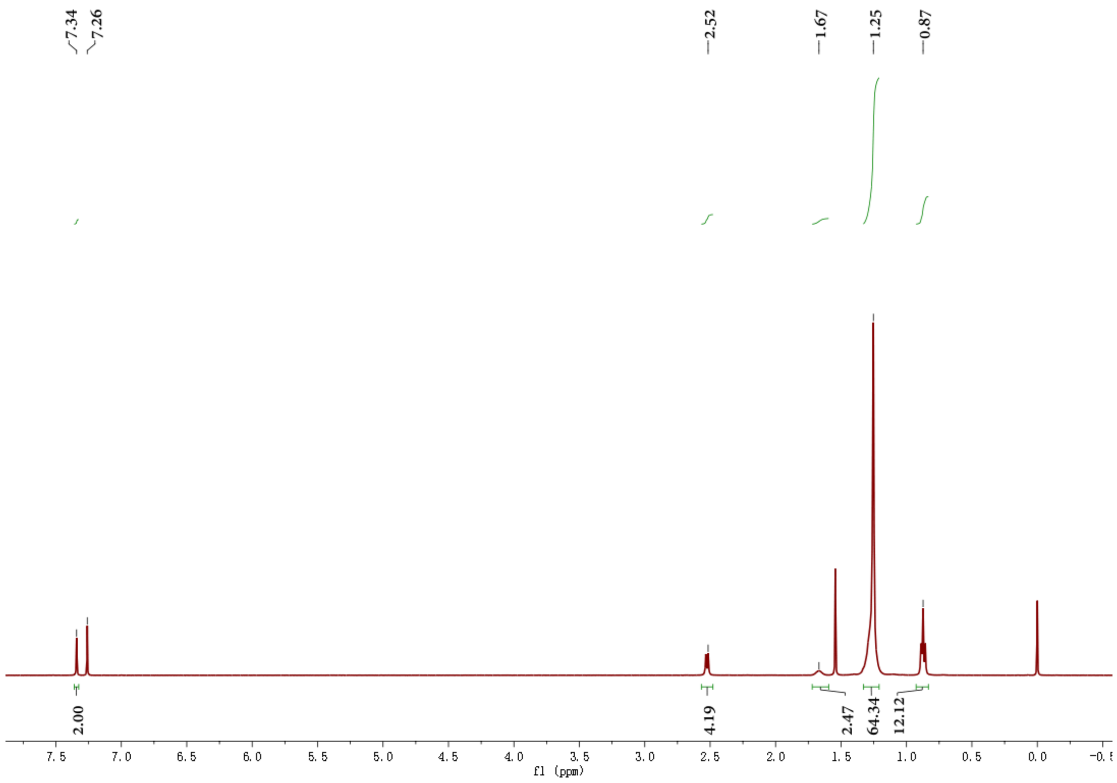

Figure S10. ^{1}H NMR spectrum of TTD2T-Br

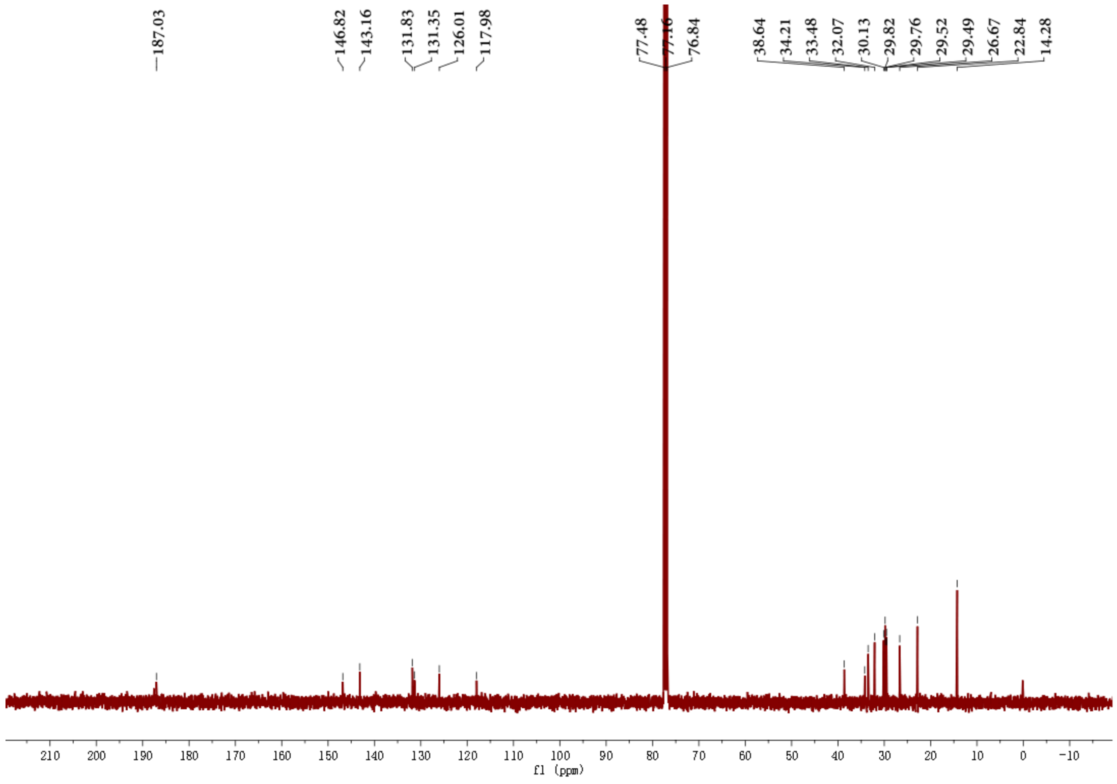

Figure S11. ^{13}C NMR spectrum of TTD2T-Br

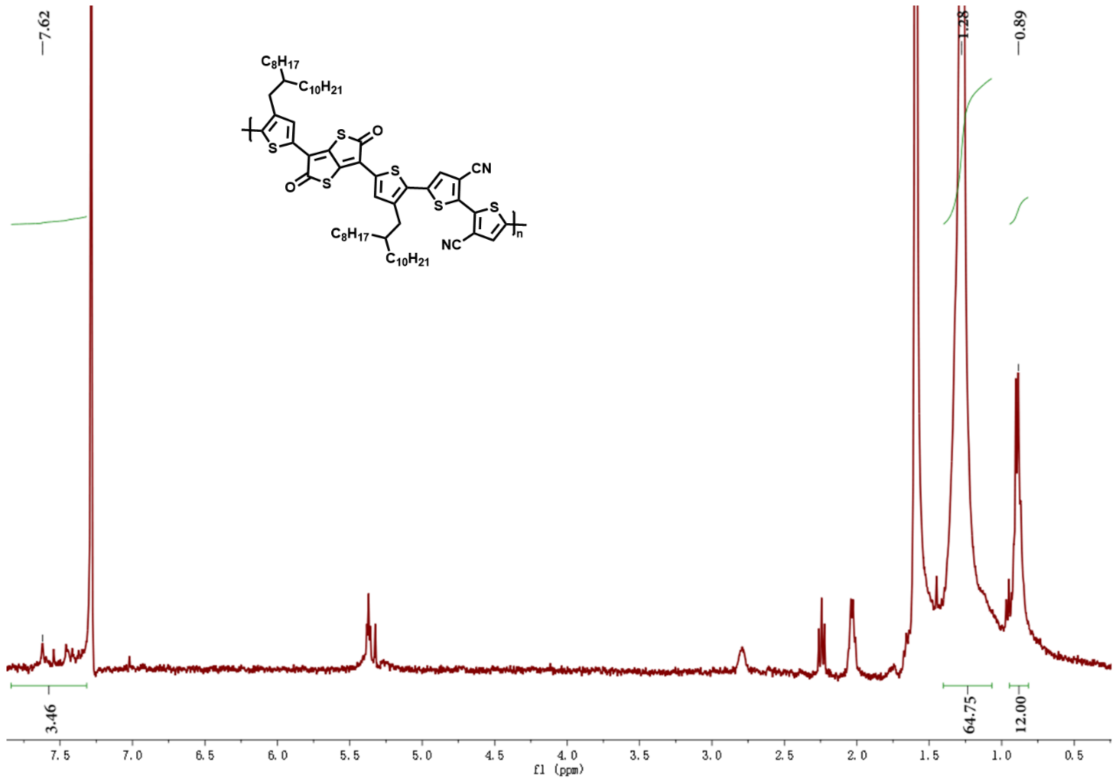

Figure S12. ^{1}H NMR spectrum of PQTTCN

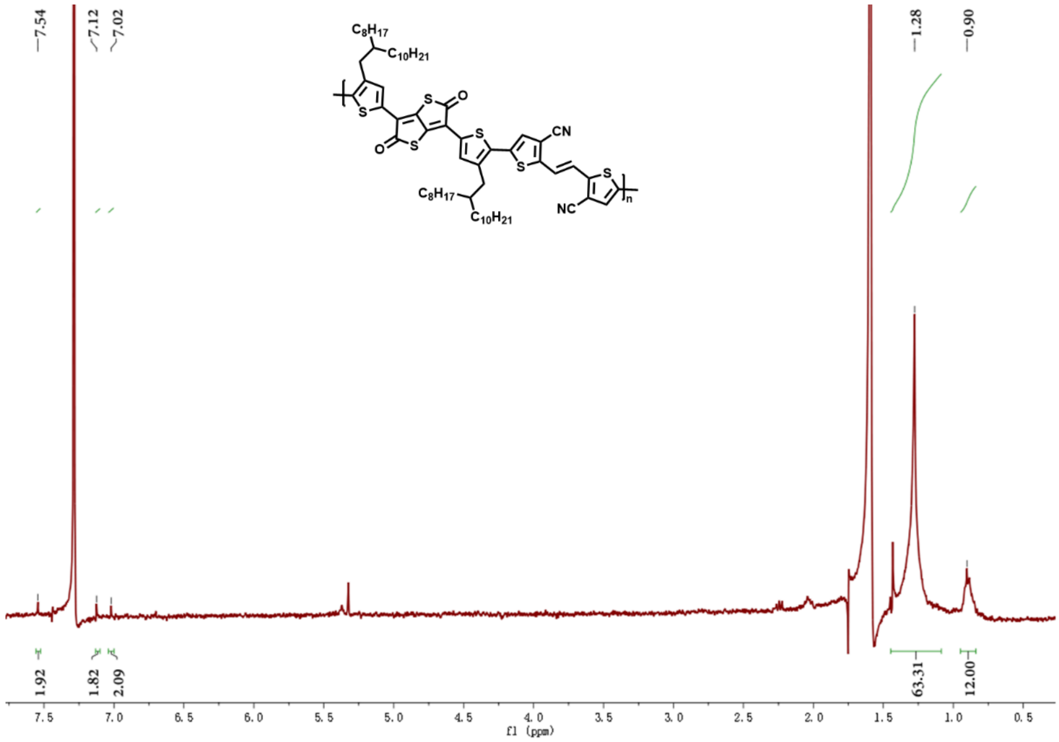

Figure S13. ^{1}H NMR spectrum of PQTVTCN

S7. References

1. Sun, W.; Gamez-Valenzuela, S.; Zhang, X.; Lee, J. W.; Zhong, Z.; Wang, P.; Ma, S.; Wang, H.; Kim, B. J.; Guo, X., A Simple Quinoid Building Block for Polymer Semiconductors with Tunable Polarity and High n-Type Thermoelectric Performance. *Angew. Chem. Int. Ed.* **2025,** *64* (28), e202501196.
2. Liao, S. F.; Chen, C. T.; Chao, C. Y., Isoindigo-dicyanobithiophene-Based Copolymer for High Performance Polymer-Fullerene Solar Cells Reaching 1.06 V Open Circuit Voltage and 8.36% Power Conversion Efficiency. *ACS Macro Lett* **2017,** *6* (9), 969-974.
3. Feng, K.; Wang, J.; Jeong, S. Y.; Yang, W.; Li, J.; Woo, H. Y.; Guo, X., High-Performance n-Type Organic Thermoelectrics Enabled by Synergistically Achieving High Electron Mobility and Doping Efficiency. *Adv. Sci.* **2023,** *10* (29), 2302629.
